# Long noncoding RNA MIAT regulates apoptosis and the apoptotic response to chemotherapeutic agents in breast cancer cell lines

**DOI:** 10.1042/BSR20180704

**Published:** 2018-07-06

**Authors:** Zainab A. Almnaseer, M. Mourtada-Maarabouni

**Affiliations:** Apoptosis Research Group, School of Life Sciences, Faculty of Natural Sciences, Keele University, Huxley Building, Keele ST5 5BG, U.K.

**Keywords:** Apoptosis, Breast, Cancer, Chemotherapy, MIAT, OCT4

## Abstract

The long noncoding RNA myocardial infarction associated transcript (MIAT) is involved in a number of diseases, including myocardial infarction and diabetic retinopathy. Emerging evidence suggests that MIAT expression levels are increased in different type of cancers, including breast cancer. In the present study, we further evaluated the role of MIAT in breast cancer and investigated the consequences of its silencing on breast cancer response to chemotherapeutic agents. Expression levels of MIAT mRNA in breast cancer were determined using TissueScan™ Breast Cancer cDNA Arrays. Breast cancer cell lines were transfected with MIAT specific siRNAs, with silencing confirmed using RT-qPCR and the effects on breast cancer cell survival and response to different apoptotic stimuli determined. MIAT transcript levels were significantly elevated in breast cancer samples. Such increase was specific to the early stages of the disease, ER, PR +ve, HER –ve, and triple negative breast cancer samples. Silencing of MIAT induced growth arrest and increased basal apoptosis. Reduced levels of MIAT augmented the apoptotic response of breast cancer cells to a wide range of apoptotic stimuli. Our results also showed that MIAT down-regulation was associated with a decrease in OCT4 mRNA, suggesting the existence of a MIAT/OCT4 regulatory loop, similar to that observed in malignant mature B cells. Taken together with the recent demonstration of oncogene characteristics, our observations suggest that MIAT plays an important role in breast tumorigenesis. Strategies to decrease MIAT expression levels may improve sensitivity to therapy in breast cancer by enhancing the apoptotic responses to conventional chemotherapies.

## Introduction

The term long noncoding RNA (lncRNA) is regularly used to describe the class of RNA transcripts longer than 200 nucleotides, which do not encode a protein [1,2]. LncRNAs have received attention due to their tissue- and developmental-specific expression patterns and their functional importance in many physiological and pathological processes [3]. Similar to mRNAs, lncRNAs are RNA polymerase II transcripts, processed via capping at the 5′ end, polyadenylated at the 3′ end and spliced. Their strong cell-type specific and temporal expression has confirmed their importance and several of these lncRNAs have now been characterized to play key roles in the control of multiple biological processes, such as gene expression, epigenetic regulation, and chromatin remodeling [3,4].

Classes of lncRNAs include long intergenic ncRNAs, natural antisense transcripts to protein coding genes, pseudogene-derived transcripts, and intronic lncRNAs [5,6]. These transcripts are known to regulate gene expression, guide chromatin-modifying complexes to specific loci, and RNA splicing by acting as signals, scaffolds, guides, or decoys [7]. Another group of lncRNAs include those that accumulate predominantly in the nucleus and serve as important components of specific nuclear bodies [8]. Some of these lncRNAs are emerging as important players in the pathogenesis of many cancers, since their expression is deregulated in tumor tissues [8]. GOMAFU/MIAT (myocardial infarction associated transcript) is of particular interest in breast cancer since its expression is up-regulated in high-grade tumors compared with low-grade ones [9].

MIAT was previously known as retinal noncoding RNA2 (RNCR2) and GOMAFU. Increasing evidence confirms the role of MIAT lncRNA in a number of cellular processes, such as the formation of nuclear bodies and neurogenic commitment [10]. In addition, MIAT lncRNA is involved in a number of diseases, including myocardial infarction [11,12], diabetic retinopathy [13], microvascular dysfunction [14], and paranoid schizophrenia [15]. Recent studies have also implicated MIAT in cancer initiation and progression [16]. MIAT was found to be up-regulated in neuroendocrine prostate cancer (NEPC) and its up-regulation was associated with Polycomb genes, which play a key role in NEPC initiation and progression [16]. Moreover, MIAT was found to be up-regulated in aggressive forms of chronic lymphocytic leukemia (CLL) and was suggested as a new biomarker for detecting the advance stages of CLL [16]. In breast cancer cells, MIAT knockdown inhibits cell proliferation and stimulates apoptosis [9]. Indeed, reduced levels of MIAT expression decreased migration and invasion in breast cancer cells and inhibited human breast tumor growth in a xenograft mouse model, suggesting that MIAT acts as an oncogene [17]. The effects of MIAT down-regulation on promoting apoptosis could have implications for breast cancer therapy, since the mode of action of many chemotherapeutic drugs is largely dependent on their interaction with apoptotic signaling pathways. While the effects of down-regulation of MIAT expression levels on breast cancer cell survival have been examined, the consequences of reduced MIAT levels for chemotherapeutic drug action in breast cancer cells have not been examined to-date.

In the present study, we have focused on the implications of reduced MIAT expression on the response to different cell death stimuli in breast cancer cells. First, we have evaluated the expression of MIAT in samples from different stages of breast cancer. Second, we have examined the effects of MIAT down-regulation on the short- and long-term survival of breast cancer cells and on the expression of OCT4, since OCT4 together with MIAT constitutes a regulatory loop in embryonic stem cells [18,19] and malignant B cells [16]. Finally, we have investigated the hypothesis that reduction in MIAT expression levels enhances the response of breast cancer cells to cell death-inducing stimuli, including chemotherapeutic drugs and ultraviolet-C (UV-C).

## Materials and methods

### Materials

MCF7, MDA-MB-231, and Hs58T cell lines were from ATCC-LGC Promochem (Teddington, U.K.). All cell culture materials and classical chemotherapeutic drugs were from Sigma-Aldrich Ltd. (Gillingham, U.K.). TissueScan qPCR Breast Cancer Disease Panels I, II, and IV (BCRT101, BCRT102, and BCRT104) were purchased from OriGene Technologies. MIAT specific siRNAs were from Qiagen (Crawley, U.K.). Negative control siRNA, TRIzol, reverse transcription reagents, and TaqMan assays were from Life Technologies Ltd. (Paisley, U.K.). SensiFast Probe Hi-ROX kit was obtained from Bioline (London, U.K.). RQ1 RNase-free DNase was from Promega (Southampton, U.K.). Nucleofector solution V was from Lonza Biosciences (Verviers, Belgium).

### Methods

#### Cell culture

The breast cancer cell lines MCF7, MDA-MB-231, and Hs58T derived from secondary stocks of cells, which had been frozen down within 2 weeks of receipt from the ATCC. Cells were cultured at 37°C in a humidified incubator in the presence of 5% CO_2_ in RPMI-1640 supplemented with 2 mM l-glutamine, 1 mM sodium pyruvate, 10 mM HEPES, 10% fetal bovine serum, and 50 μg/ml gentamicin. Cells were replaced with fresh stocks after a maximum culture period of 2 months.

#### RNA interference by siRNA

Cells were nucleofected with Qiagen siRNAs using programmes E-014 for MCF7 and X-013 for MDA-MB-231 and Hs58T, as described previously [20]. Three different MIAT siRNAs targeting different locations on exon 5 were used termed M1 (Product code # SI04314919; target site 6488-6508), M2 (# SI04287423; target site 7222-7242), and M3 (# SI00582799; target site 9735-9755). Control cells received a negative control siRNA (NC; code AM4611). Following nucleofection, cells were plated in 3 ml of complete RPMI-1640 medium in six-well plates.

#### Induction of cell death

At 72 h post-siRNA nucleofection, cells were trypsinized, sampled for RNA extraction, counted using a hemocytometer following staining with Trypan Blue (0.4% w/v; Sigma), and then seeded (0.8 × 10^5^ cells/ml) into 12-well plates. In some experiments, ultraviolet-C (UV-C) irradiation (dose: 20 J/m^2^) was performed prior to plating, while controls were mock-irradiated [21]. For drug treatments, cells were cultured for 20 h, before addition of 5-fluorouracil (5-FU, 175 μM), docetaxel (10 µM), Nutlin-3 (5 µM), mitoxantrone (50 µM), or vehicle (0.25% v/v dimethyl sulfoxide). Cells were cultured for 48 h before assessment of cell viability and apoptosis.

#### Cell survival assays and cell cycle analysis

Apoptosis level was determined by flow cytometry using a Muse annexin V and dead cell assay kit, according to the manufacturer’s protocol (Merck Millipore, Darmstadt, Germany) [20]. Apoptosis was also measured by determining caspase activity using CaspaTag Pan-Caspase In Situ Assay Kit, according to the supplied instructions (Millipore, Watford, U.K.).

Cell viability was determined using a commercial Cell Count and Viability Kit and a Muse flow cytometer (Merck Millipore, Darmstadt, Germany) [20]. Clonogenic assays were performed to assess long-term survival. Transfected cells were replated in culture medium supplemented with 10% (v/v) cell-conditioned medium in six-well plates and cultured for 3 weeks. The number of the colonies was determined after staining with Crystal Violet stain (0.5% (w/v) in methanol) for 10 min.

Cell cycle analysis was carried using nuclear propidium iodide (PI)-staining procedure and flow cytometry [20]. Transfected cells were harvested 48 h post-transfection and replated in fresh medium at 2 × 10^5^ cells/well in six-well plates. Following incubation for 24 h, 1 million cells were washed in PBS before resuspending the pellet in 200 μl of PBS. Cells were then fixed in 1 ml of ice-cold 70% ethanol/30% PBS and stored at −20°C for at least 3 h prior to cell cycle analysis. Fixed cells were centrifuged for 5 min at 1500 rpm. The supernatant was discarded and the cell pellet was resuspended in 200 μl of Muse Cell Cycle Reagent (Merck Millipore # MCH100106). Cells were incubated for 30 min in the dark and data acquisition was carried out using the Millipore Muse cell analyzer [20].

#### Real-time RT-PCR (RT-qPCR)

Total RNA from 10^7^ cells was isolated using Trizol according to the manufacturer’s instructions. Five micrograms of RNA was reverse transcribed using SuperscriptTM II RNase H reverse transcriptase and random primers as described previously [21]. TaqMan gene expression assays (assay codes Hs99999901_m1 for 18S and Hs00402814_m1 for MIAT) were employed. Assays contained 10 ng sample cDNA or 0.2–30 ng standard cDNA (prepared from cDNA from MCF7, MDA-MB-231, and Hs58T breast cancer cells). The ABI Prism 7000 sequence detection system was used to measure real-time fluorescence. Input amounts of samples were calculated from their respective threshold cycle (*C*_T_) values, using standard curves generated with each assay. Data were expressed relative to 18S rRNA.

#### Human tissue samples

Real-time PCR was also performed on TissueScan™ Cancer and Normal Tissue cDNA Arrays using Breast Cancer cDNA Array I, II, and IV covering cDNA from 16-normal, 23-Stage I, 58-II, 42-III, and 5-IV samples, whose clinical and pathological features are freely available at the following address: http://www.origene.com/qPCR/Tissue-qPCR-Arrays.aspx. Standard curve of threshold cycle (*C*_T_) value versus log input standard cDNA was constructed by linear regression, and the equation of the line was used to calculate input amounts of samples from their respective *C*_T_ values. Data were expressed relative to 18S.

### Statistical analysis

Data are presented as mean ± standard error of the mean (S.E.). Data were analyzed by one-way analysis of variance, using Bonferroni’s multiple comparison test (MCT). Data from the Breast Cancer cDNA Arrays were analyzed by Mann–Whitney rank sum test between normal and cancer samples. Comparison between normal specimen and different stages or subtypes of cancer was carried out using Kruskal–Wallis one-way analysis of variance (ANOVA) on ranks, followed by Dunn’s test for comparisons against the control group. GraphPad Prism v4.03 was used to construct the graphs and perform statistical analyses.

## Results

### Expression of MIAT lncRNA in breast cancer

**Figure 1:**
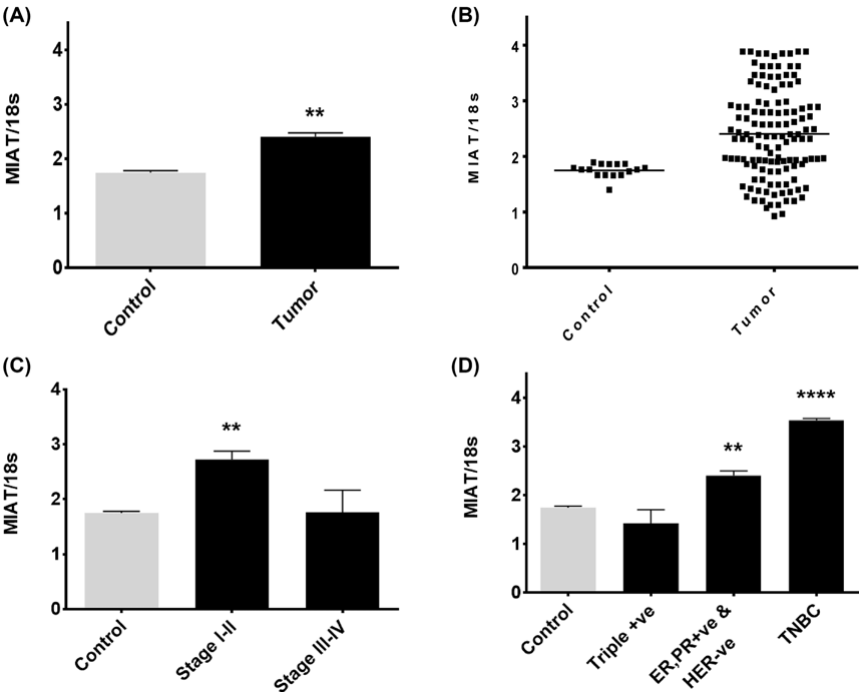
MIAT expression levels in breast cancer The level of MIAT lncRNA was determined in 128 tumor samples and compared with 16 normal breast tissue samples. (**A**) MIAT expression showed significant increase in the full data sets of breast tumors compared with normal. (**B**) Corresponding scattergrams of these data are shown. (**C**) Analysis of the data revealed that there is a highly significant up-regulation of MIAT lncRNA in stage I–II disease. (**D**) Stratification of MIAT expression levels according to the molecular subtypes showed variation in MIAT expression. MIAT expression was not changed in the triple positive (ER, PR, and HER2 positive) samples, significantly increased in ER, PR +ve, HER –ve, and TNBC breast cancer subtypes (Mann–Whitney Rank Sum Test (A and B) and Kruskal–Wallis one-way ANOVA on ranks (C and D); **P<0.01 and ****P< 0.0001; *n*=16 (normal), 128 (breast adenocarcinoma specimens)).

Previous studies have reported an increased in MIAT expression levels in high-grade breast tumors compared with low-grade ones [9]. MIAT expression was also found to be up-regulated in ER, PR, and Her2 positive tumor tissues [9]. To further investigate whether the mRNA expression levels of MIAT exhibit changes in breast cancer, we utilized breast cancer cDNA arrays (BCRT101, BCRT102, and BCRT104) from OriGene Inc and determined MIAT expression levels in breast cancer and normal tissues. Real-time PCR was performed using TaqMan gene expression assays targeting MIAT and 18S, which was used as an endogenous control gene. The results revealed that the overall mean of MIAT expression level was significantly higher in breast tumor compared with the mean of MIAT expression levels in normal breast tissue (Figure 1A). However, scattergram analysis of these data demonstrated heterogeneity (Figure 1B) and further analysis revealed an increased in MIAT expression level in stage I–II breast samples but not in stage II–IV (Figure 1C). Stratification of patients into groups according to molecular subtypes showed that compared with the normal breast samples MIAT expression was not changed in the triple positive (ER, PR, and HER2 positive) samples, but was significantly increased in ER, PR +ve, HER –ve breast cancer subtypes and in triple negative breast cancer (TNBC) (Figure 1D). The increase in MIAT levels was more enhanced (2-fold) in TNBC samples.

### Effect of MIAT silencing on OCT4 mRNA expression and on the survival of breast cancer cells

To examine the effects of reduced MIAT expression on breast cancer cell survival, MIAT siRNAs were employed to silence endogenous MIAT expression in two cell lines, MCF7 and MDA-MB-23. Three different siRNAs were employed to reduce the possibility of ‘off-target’ effects. The influence of MIAT silencing on the short- and long-term survival of breast cancer cells was examined under basal conditions.

**Figure 2:**
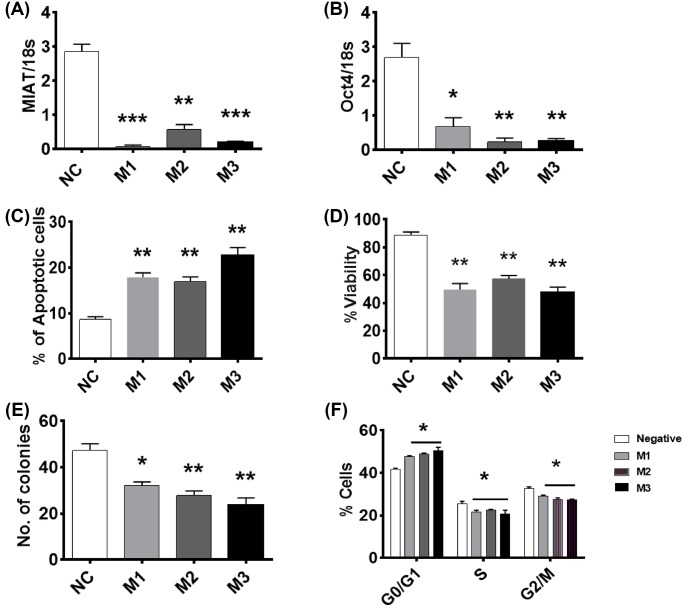
Effect of MIAT silencing on the survival of MCF7 cells MCF7 cells (*n*=4 cultures) were transfected with the indicated MIAT siRNA or negative control (NC) siRNA and harvested at 72 h post-transfection. Cells were replated for assessment of cell survival after a further 48 h. (**A**) At 72 h post-transfection, RT-qPCR analysis confirmed decreased cellular MIAT levels in cells treated with MIAT siRNAs. (**B**) Cells transfected with MIAT siRNAs expressed less OCT4 mRNA as confirmed with RT-qPCR. (**C**) Silencing of MIAT enhanced basal apoptosis. (**D**) Short-term viability was reduced 48 h post replating. (**E**) Colony formation in long-term clonogenic assays was significantly decreased. (**F**) Cell cycle analysis revealed that MIAT silencing caused an elevation in the percentage of cells in G0/G1 phase; *P<0.05, **P<0.01, and ***P<0.001 versus cells transfected with NC siRNA (one-way ANOVA and Bonferrroni’s MCT).

In MCF7 cells, all three siRNAs reduced MIAT transcript levels by up to 70–85% compared with control levels (Figure 2A). OCT4 is a transcription factor reported to promote MIAT expression and a positive correlation between MIAT and OCT4 mRNA expression levels has been reported [16]. Therefore, the effects of reduced MIAT expression levels on the expression of OCT4 mRNA were also determined. Down-regulation of MIAT was found to be associated with a decrease in OCT4 mRNA levels, with OCT4 transcript levels found to be 65–80% lower than that of control cells (Figure 2B), indicating the positive relationship between MIAT and OCT4 mRNA expression. MIAT down-regulation caused a significant increase in basal apoptosis levels and a reduction in both short-term survival and the clonogenic activity (Figure 2C–E). Analyzing the cell cycle profile revealed that that MIAT silencing causes an increase in the percentage of cells in G0/G1, and a concomitant significant decrease in the percentage of cells in S and G2/M phase (Figure 2F).

**Figure 3:**
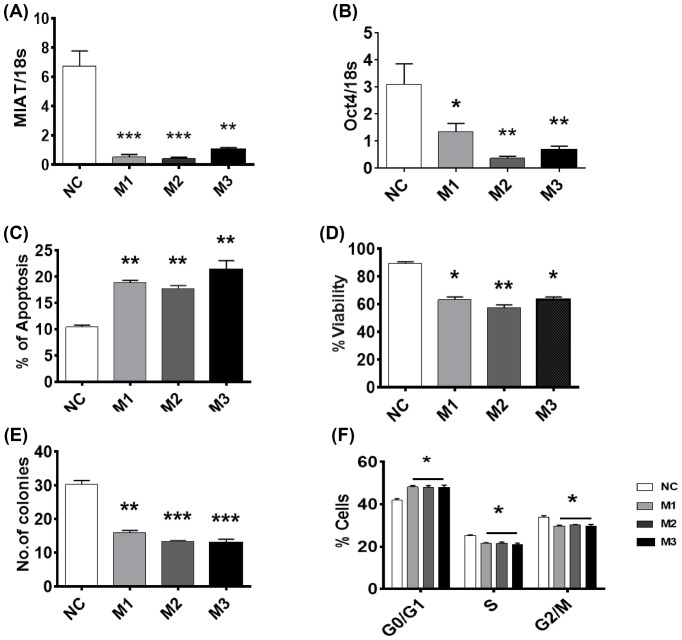
Effect of MIAT siRNAs on the survival of MDA-MB-231 cells MDA-MB-231 cells were transfected with the indicated MIAT siRNA or negative control (NC) siRNA and harvested at 72 h post-transfection (*n*=4 cultures). (**A**) At 72 h post-transfection, RT-qPCR analysis confirmed decreased cellular MIAT levels. (**B**) mRNA levels of OCT4 were also reduced in MIAT transfected cells. (**C**) Apoptosis levels measured 48 h post replating showed that silencing of MIAT enhanced basal apoptosis. (**D**) Transfection of MIAT siRNAs caused a reduction in short-term viability. (**E**) Clonogenic ability was reduced in the cells transfected with MIAT siRNAs. (**F**) Cell cycle analysis revealed that MIAT silencing caused an elevation in the percentage of cells in G0/G1 phase; *P<0.05, **P<0.01, and ***P<0.001 versus cells transfected with NC siRNA (one-way ANOVA and Dunnett’s MCT).

Consistent with these observations, MIAT down-regulation (80% decrease compared with control) in TNBC MDA-MB-231 cells (Figure 3A) was associated with a 65–80% decrease in OCT4 levels (Figure 3B) and a reduction in short-term survival and clonogenic activity (Figure 3D,E). Basal apoptosis levels were also enhanced by MIAT silencing (Figure 3C). Cell cycle analysis showed that similar to observations in MCF7 cells, silencing of MIAT in MDA-MB-231 increased the percentage of cells in G0/G1, with an associated decrease in the percentage of cells in S and G2/M phase (Figure 3F).

### The effect of MIAT silencing on UV-C and chemotherapeutic drug-induced cell death in breast cancer cells

**Figure 4:**
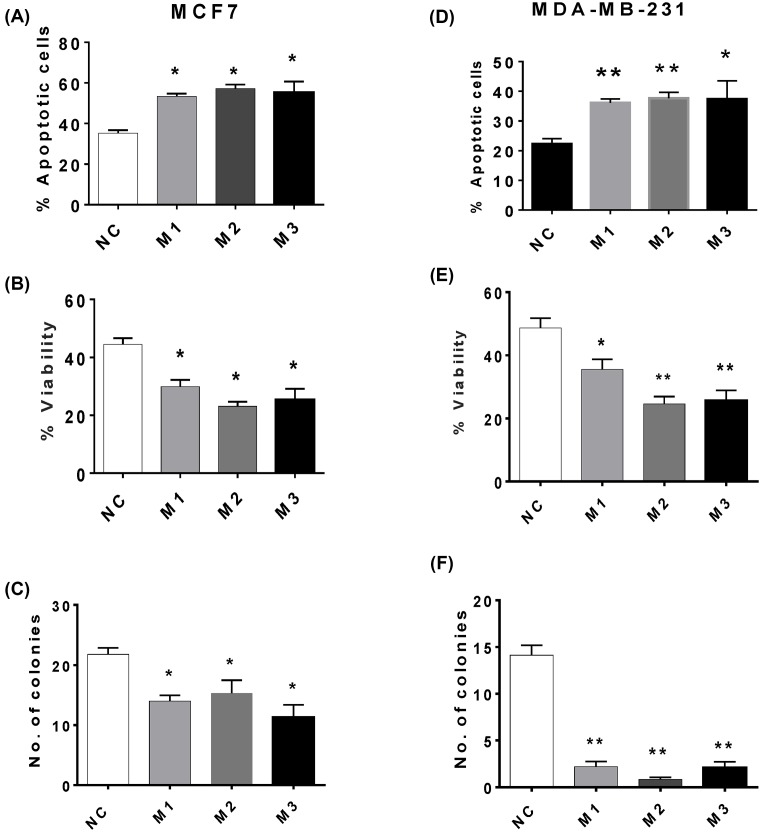
Effect of MIAT siRNAs on UV-C induced cell death in MCF7 and MDA-MB-231 cells Breast cancer cells (*n*=4 cultures) were transfected with the indicated MIAT siRNA or negative control (NC) siRNA and harvested at 72 h post transfection. Cells were exposed to UV-C irradiation and cell survival parameters were determined 48 h post-treatment. For all figure parts, MCF7 results are displayed in the left-hand panel and MDA-MB-231 cells in the right-hand panel. (**A**) and (**D**) Reduced levels of MIAT enhanced UV-C-induced apoptosis. (**B**) and (**E**) MIAT silencing increased UV-C-induced loss of cell survival. (**C**) and (**F**) MIAT silencing also promoted UV-induced loss of clonogenic activity; *P<0.05 and **P<0.01 versus cells transfected with NC siRNA (one-way ANOVA and Dunnett’s MCT).

**Figure 5:**
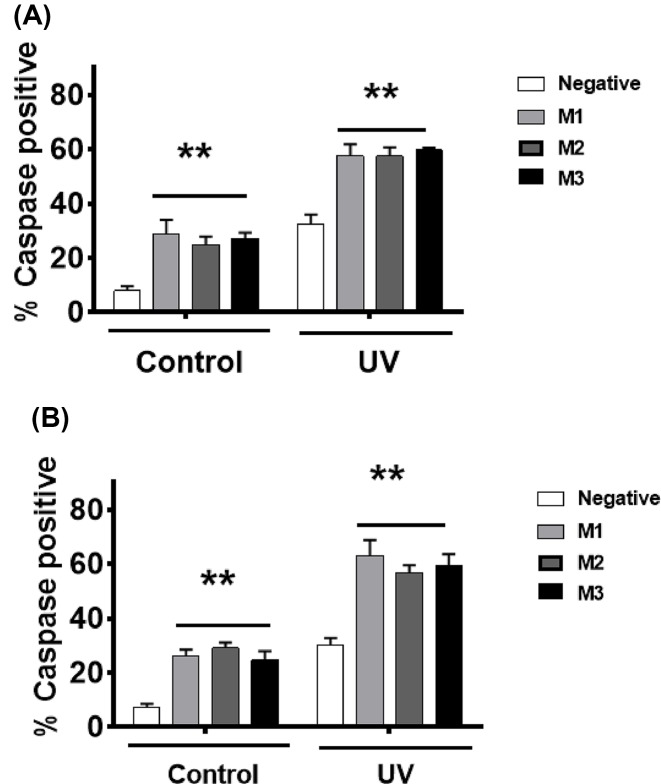
MIAT silencing promotes caspase activation in breast cancer cells Breast cancer cells were transfected with the indicated MIAT siRNA or negative control (NC) siRNA. At 72 h post-transfection, cells were trypsinized and treated ± UV-C light (20 J/m^2^). Cells were incubated for 24 h before the assessment of caspase activity. Percentage of cells containing activated caspases was determined using fluorescence microscopy. (**A**) MIAT silencing caused an enhanced caspase activity in MCF7 breast cancer cells. (**B**) Transfection of MIAT siRNAs caused an enhanced caspase activity in MDA-MB-231 breast cancer cells; **P<0.01 versus cells transfected with NC (one-way ANOVA and Bonferrroni’s MCT; *n*=4).

**Figure 6:**
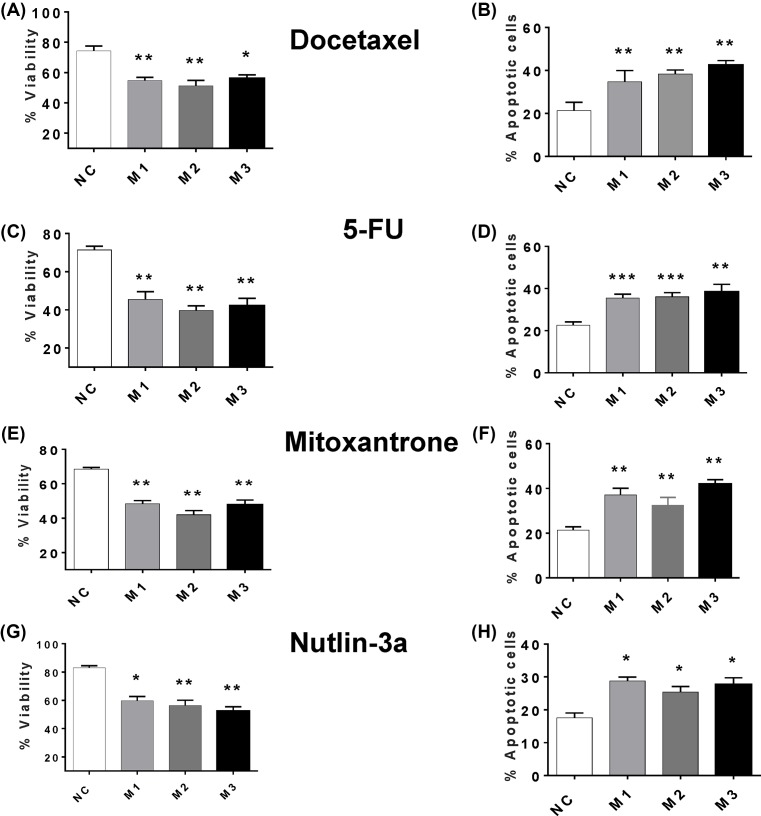
Effect of MIAT siRNAs on the survival of MCF7 cells after treatment with chemotherapeutic drugs MCF7 cells (*n*=4 cultures) were transfected with MIAT siRNA or negative control (NC) siRNA and after 72 h, treated with either 5-FU (175 μM), docetaxel (10 μM), nutlin-3 (5 μM), mitoxantrone (50 μM) or vehicle (0.25% dimethyl sulfoxide). Following 48 h incubation, cell viability was determined by flow cytometry on harvested cells and apoptosis was determined by annexin V staining and flow cytometry. For all figure parts, cell viability is displayed in the left-hand panel and apoptosis in the right-hand panel. (**A** and **B**) MIAT silencing enhanced the loss of viability and apoptosis induced by docetaxel. (**C** and **D**) Similar effects were observed with 5-FU. (**E** and **F**) Cells transfected with MIAT siRNAs exhibit enhanced loss of cell viability and an increased in apoptosis level after mitoxantrone treatment. (**G** and **H**) Loss of viability and induction of apoptosis by nutlin-3a were enhanced in MIAT siRNA transfected cultures; *P<0.05, **P<0.01, and ***P<0.001 versus cells transfected with NC siRNA (one-way ANOVA and Bonferrroni’s MCT).

**Figure 7:**
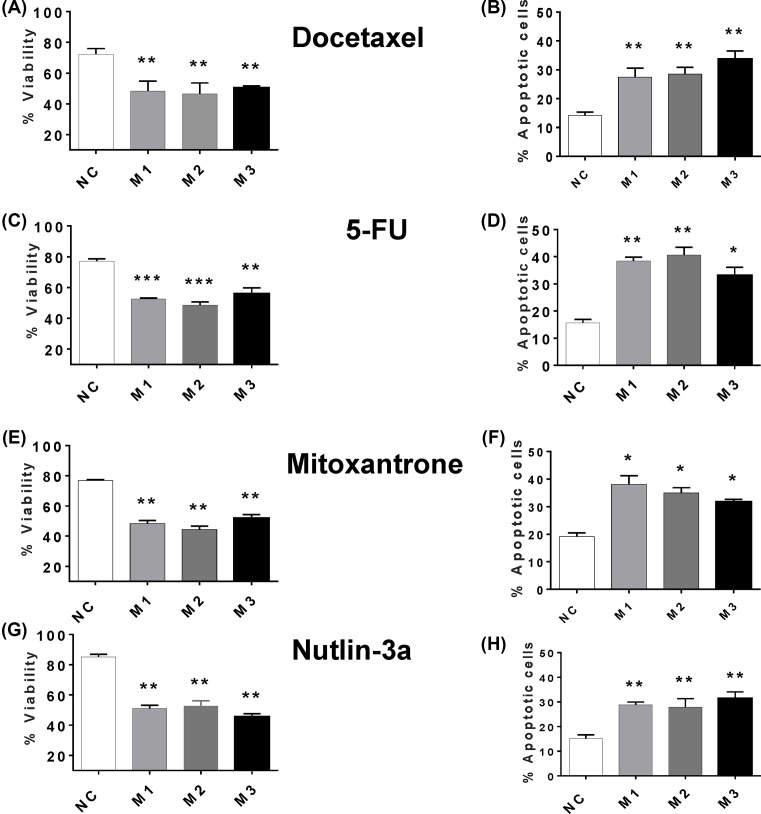
Effect of MIAT silencing on chemotherapeutic drug-induced death of MDA-MB-231 TNBC cell line MDA-MB-231 cells (*n*=4 cultures) were transfected with the indicative MIAT siRNA or negative control (NC) siRNA. At 72 h post-transfection, cells were replated and treated with either 5-FU (175 μM), docetaxel (10 μM), nutlin-3 (5 μM), mitoxantrone (50 μM) or vehicle (0.25% dimethyl sulfoxide). After 48 h incubation, cell viability was determined by flow cytometry and apoptosis was determined by annexin V staining and flow cytometry. For all figure parts, cell viability is displayed in the left-hand panel and apoptosis in the right-hand panel. (**A**) and **B**) MIAT silencing enhanced the loss of viability and apoptosis caused by docetaxel. (B and **C**) Cells transfected with MIAT siRNAs exhibit enhanced loss of cell viability and an increased in apoptosis level after 5-FU treatment. (**E** and **F**) MIAT transfected cells showed decreased cell viability and enhanced apoptosis after mitoxantrone treatment. (**G** and **H**) Same results were obtained with nutlin; *P<0.05, **P<0.01, and ***P<0.001 versus cells transfected with NC siRNA (one-way ANOVA and Bonferrroni’s MCT).

When MCF7 cells were irradiated with UV-C light, MIAT knockdown enhanced apoptosis levels (Figure 4A) and the consequent decrease in short- (Figure 4B) and long-term (Figure 4C) culture viability. Consistent with these observations, MIAT knockdown in MDA-MB-231 cells stimulated apoptosis induction by UV-C irradiation (Figure 4D), as well as the associated losses in culture viability (Figure 4E) and clonogenic activity (Figure 4F). It is worth noting that the extent of MIAT knockdown-mediated stimulation of apoptosis in response to UV-C irradiation tended to be similar to that seen in the absence of any apoptotic stimuli, suggesting that there is an additive rather than synergistic effect of MIAT silencing on UV-induced cell death. Further experiments involving the assessment of caspase activity confirmed that MIAT knockdown in both cell lines MCF7 and MDA-MB-231 is specifically associated with higher levels of apoptosis in both basal conditions and after UV-C treatment (Figure 5A,B). Similar results were obtained upon treatment with the chemotherapeutic drugs, docetaxel, 5-fluoracil, mitoxantrone, and nutlin 3a, where MIAT silencing in MCF7 (Figure 6A–H) and MDA-MB-231 (Figure 7A–H) enhanced the apoptotic response and the loss of culture survival of these cells. Again the effects of MIAT silencing were additive to the effects of apoptotic stimuli on the negative cells.

### MIAT silencing also induces apoptosis in triple-negative Hs58T cells

**Figure 8:**
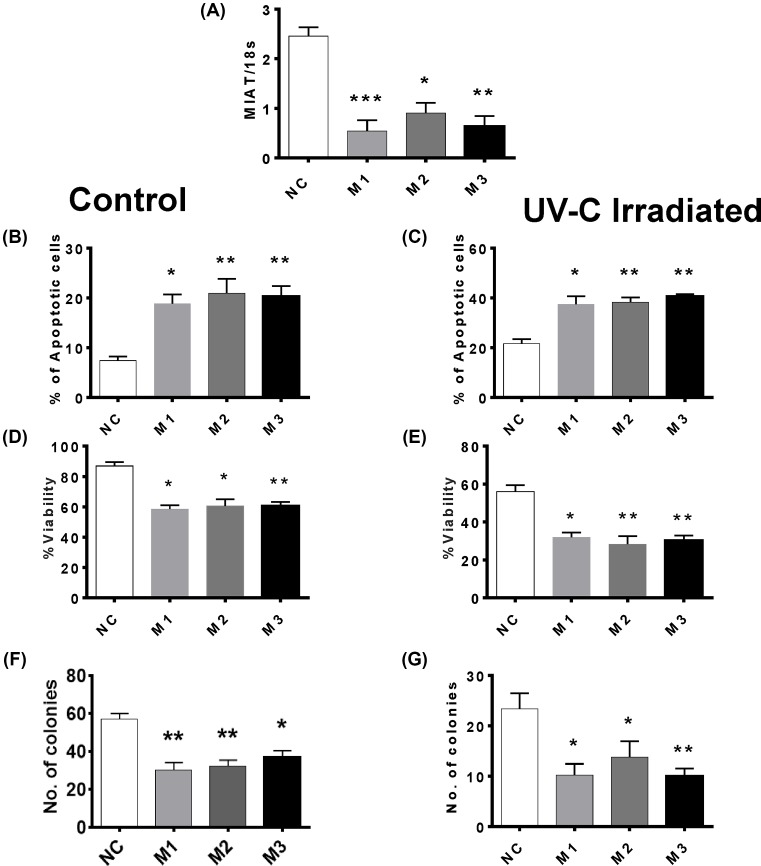
Effect of MIAT knockdown on the basal survival and UV-C induced cell death in triple-negative Hs58T breast cancer cells Hs58T cells (*n*=4 cultures) were transfected with the indicated MIAT siRNA or negative control (NC) siRNA. Cells were harvested at 72 h post-transfection and were treated ± UV-C irradiation and replated for a further 48 h before the assessment of cell survival. (**A**) RT-qPCR analysis confirmed decreased cellular MIAT levels at 72 h post-transfection in cells treated with MIAT siRNAs. (**B**) Basal apoptosis levels were increased in MIAT siRNA transfected cells. (**C**) Transfection with MIAT siRNAs enhanced UV-C-induced apoptosis. (**D**) Correspondingly, cell viability was also reduced in cells transfected with MIAT siRNAs. (**E**) Loss of viability caused by UV was more enhanced in the cells transfected with MIAT siRNAs. (**F**) MIAT silencing induces loss of clonogenic activity. (**G**) UV-C-induced loss of colony forming ability was more enhanced in MIAT siRNA transfected cells; *P<0.05, **P<0.01, and ***P<0.001 versus cells transfected with NC siRNA (one-way ANOVA and Bonferrroni’s MCT).

To further confirm our observations that silencing MIAT is associated with an increase in apoptosis levels in breast cancer cells, we examine the influence of MIAT silencing on the cell survival of another TNBC cell line Hs58T. Hs58T cells were transfected with the three MIAT specific siRNAs and cell survival was examined under basal conditions and after apoptosis induction by UV-C irradiation. Decreased MIAT levels (Figure 8A) under basal conditions were associated with an increase in basal apoptosis (Figure 8B) and a decrease in both short-term viability (Figure 8D) and long-term survival (Figure 8F). MIAT knockdown also enhanced apoptosis induction by UV-C irradiation (Figure 8C) as well the associated loss of short- and long-term cell survival (Figure 8E,G). Thus, as for MCF7 and MDA-MB-231, MIAT silencing leads to an increase in apoptosis and reduction in Hs58T cell survival.

## Discussion

MIAT was originally identified in the neurons of the mouse retina and was later found to be highly expressed in the nervous system throughout development [22]. Its role was subsequently confirmed in a number of cellular processes, including the formation of nuclear bodies [23], regulation of mammalian retinal cell differentiation [24], and neurogenic commitment [25]. In addition, MIAT was shown to be involved in a number of diseases, such as myocardial infarction [10,11], diabetic retinopathy [12], microvascular dysfunction [13], and paranoid schizophrenia [14]. Evidence implicating MIAT in cancer is now emerging and MIAT is selectively up-regulated in neuroendocrine prostate cancer [15] and in primary leukemic cells from patients with aggressive forms of CLL [16].

A role of MIAT lncRNA in breast cancer is also emerging; studies have reported an increased MIAT expression levels in advanced breast tumors, where its overexpression was shown to be associated with lymph node metastasis [17]. Using gene expression profiling of breast cancer tissues, the present study confirms that MIAT expression level is significantly increased in breast cancer, specifically in the early stages of the disease (stage I and II), suggesting that MIAT overexpression is important in the early stages of breast oncogenesis and may play a key role throughout the development of malignancy. On the other hand, there was no significant increase in MIAT levels in breast cancer samples in the late stages of the disease, this could be due to the small sample size. Unlike previous studies that showed an increased MIAT expression levels in the triple positive (ER, PR, and Her2 positive) tumor tissues [9], our results showed that MIAT expression was not changed in the triple positive breast cancer samples. However, MIAT expression levels showed significant increase in ER, PR +ve, HER –ve, and the TNBC breast cancer subtypes. The increase in MIAT expression levels was more enhanced in the TNBC samples, consistent with the observations that MDA-MB-231 TNBC cell line showed the highest expression levels of MIAT when compared with other breast cancer cell lines [17]. Together, the data suggest that increased MIAT expression levels might be associated with an increase in the metastatic ability of breast cancer.

Functional studies have previously reported that MIAT regulates cancer cell survival, with decreased MIAT expression levels inhibiting cell proliferation in breast cancer cells [9,17] and malignant B cells [16]. Our results further support such observations and confirm that MIAT down-regulation is associated with enhanced basal apoptosis levels and a decrease in short- and long-term survival of different types of breast cancer cells. Our study further extends these findings and shows, for the first time, that MIAT not only regulates basal apoptosis levels, but also modulates the response of breast cancer cells to a range of apoptotic stimuli. Low levels of MIAT were consistently associated with increased cell death in response to both physical and chemical stimuli. MIAT silencing enhanced breast cancer cell response to a diverse range of apoptotic stimuli acting via different mechanisms, including DNA damage (UV-C irradiation), inhibition of mouse double minutes clone 2 (MDM2) (nutlin-3a), microtubule stabilizing (docetaxel), inhibition of topoisomerase II (mitoxantrone), and inhibition of thymidylate synthase (5-fluorouracil), indicating that MIAT participates in a late common step that can connect the different apoptotic pathways activated by these agents. Such observation is characteristic of the actions of key apoptosis regulators such as inhibitor of apoptosis protein family and pro- and antiapoptotic members of the Bcl-2 family, for which their differential expression is key in determining cancer cell sensitivity to cell death induced by different stimuli [26,27]. It is therefore important to examine the functional relationships associating MIAT with these key regulators of apoptosis execution. Indeed, reduced levels of MIAT in PC9 lung cancer cells were recently found to be associated with a decrease in Bcl-2 and an increase in Bax expression levels [28], providing further evidence that associates reduced MIAT levels with an enhanced responses to a diverse range of cell death stimuli and raising the possibility that increased MIAT levels might contribute to the development of drug resistance in metastatic disease.

OCT4 is a transcription factor that contributes with MIAT in forming a regulatory feedback loop that regulates cell fate [16,18,29,30]. GOMAFU, the mouse homolog of MIAT, has been shown to bind to OCT4 gene leading to an increase in its expression and OCT4 was also reported to positively regulate GOMAFU transcription in mouse ES cells [18]. The existence of such a regulatory feedback loop was further validated in aggressive chronic lymphocytic leukemia, where MIAT and OCT4 were found to increase survival and proliferation and suppress apoptotic cell death in malignant mature B cells and in cancer stem cell-like cells [16]. OCT4 overexpression increases cell proliferation and inhibits apoptosis induction in myeloid cells with mature B-cell phenotype and in cancer stem cell-like cells, whereas its down-regulation increases cell death and inhibits proliferation [16,31,32]. The present work also supports the existence of an MIAT and OCT4 axis involved in the regulation of breast cancer cell survival.

Consistent with others [9], our studies showed that MIAT silencing altered the cell cycle profile causing an increase in the percentage of cells in G1 phase and a concomitant decrease in the cells in G2/M phase. These effects might be due to the decreased levels of OCT4 in these cells. Suppression of OCT4 expression leads to the activation of the cyclin-dependent kinase inhibitor p21 [31]. Active p21 acts as tumor suppressor by promoting cell cycle arrest in G1 and therefore inhibiting cell cycle progression [31]. Besides its role as coactivator for OCT4 mRNA expression, MIAT acts as a competing endogenous RNA (ceRNA), where it forms a regulatory feedback loop with miRNA-150-5p and vascular endothelial factor [18,33,34]. MIAT also acts as a ceRNA in human lens epithelial cells (HLECs), where it forms a feedback loop with Akt and miR-150-5p to regulate HLEC function and survival [35].

In a wide variety of physiological settings, the balance between apoptosis and cell proliferation is important in tissue development, regulation of cell number, and removal of unwanted and harmful cells. Consequently, dysfunctional apoptosis and uncontrolled cell proliferation can result in a wide variety of diseases, including cancer [35–37]. Defective apoptosis is also associated with resistance to conventional chemotherapies since the actions of many anticancer chemotherapeutic agents depend on their interactions with apoptotic signaling pathways [25,26,36–41]. The present study shows that MIAT lncRNA is up-regulated in breast cancer and in particular in TNBC, suggesting that MIAT may act as oncogene. The findings that reduced levels of MIAT enhanced both basal apoptosis of breast cancer cells and their response to a range of apoptotic stimuli could therefore have important clinical significance in relation to the response of breast tumors to cytotoxic therapy. Given the observations that comparable effects of MIAT silencing were witnessed in triple positive and triple negative breast cancer cells, whilst the expression levels of MIAT varied in relation to tumor grading by clinical stages, further studies using the MCF10 series representing different cancer stages should shed further light on the role of MIAT in breast cancer progression.

## Conclusion

Our findings indicate that MIAT expression level is increased in breast cancer. MIAT was found to be a key regulator of breast cancer cell survival. Reduced levels of MIAT inhibited short- and long-term survival, increased basal apoptosis, and enhanced breast cancer cell apoptotic response to a range of apoptotic stimuli in an additive way. Our results suggest that approaches to decrease MIAT levels in breast tumors, coupled to conventional chemotherapeutic strategies, may prove beneficial in improving breast cancer patient outcomes. Such an approach may be particularly beneficial for TNBC, which often exhibits resistance to conventional chemotherapies. Our current findings in breast cancer cell lines and recent findings in lung cancer [27] may be relevant to other cancers characterized by increased MIAT levels, including ovarian cancer [42] and gastric cancer [43]. Future studies will determine the potential use of MIAT as biomarker and specific therapeutic target.

## Abbreviations

ceRNA: competing endogenous RNA
CLL: chronic lymphocytic leukemia
5-FU: 5-fluorouracil
HLEC: human lens epithelial cell
LncRNA: long noncoding RNA
MCT: multiple comparison test
MIAT: myocardial infarction associated transcript
NEPC: neuroendocrine prostate cancer
Oct4: octamer-binding transcription factor 4
RNCR2: retinal noncoding RNA2
TNBC: triple negative breast cancer
